# Baseline value and longitudinal kinetics of circulating nucleosomes during neo-adjuvant chemotherapy in newly diagnosed ovarian cancer: Results from a GINECO/GINEGEPS study of the randomized phase II CHIVA trial

**DOI:** 10.1016/j.tranon.2026.102896

**Published:** 2026-07-01

**Authors:** Pauline Corbaux, Olivier Colomban, Gaelle Lescuyer, Isabelle Ray-Coquard, Gaëtan De Rauglaudre, Florence Joly, Cyril Abdeddaim, Pierre Combe, Cyriac Blonz, Guillaume Bataillon, Jérôme Meunier, Jérôme Alexandre, Dominique Berton, Marie-Christine Kaminsky, Diana Bello Roufai, Alexandra Leary, Laurence Venat, Nadine Dohollou, Christophe Louvet, Coriolan Lebreton, Sophie Abadie-Lacourtoisie, Jean-Pierre Lotz, Laure Favier, Michel Fabbro, Nathalie Bonichon-Lamichhane, Jean-Emmanuel Kurtz, Philippe Follana, Eric Pujade-Lauraine, Lea Payen, Benoit You

**Affiliations:** a Center for Innovation in Cancerology of Lyon (CICLY) EA 3738, Hospices Civils of Lyon, Faculty of Medicine and Maieutic Lyon Sud, Claude Bernard University Lyon I, 69921, Oullins, France; b Medical Oncology, Institut de Cancérologie et d'Hématologie Universitaire de Saint-Étienne (ICHUSE), Centre Hospitalier Universitaire de Saint-Etienne, France; c GINECO (Groupe d’Investigateurs Nationaux pour l’Etude des Cancers de l’Ovaire), France; d Department of Biochemistry and Molecular Biology, Lyon-Sud Hospital, Hospices Civils de Lyon, Pierre-Bénite, France; e Centre Léon Bérard, Lyon, France; f Institut Sainte-Catherine, Avignon, France; g Centre François Baclesse, Caen, France; h Centre Oscar Lambret, Lille, France; i Hôpital Européen Georges Pompidou, Paris, France; j Hôpital Privé du Confluent, Nantes, France; k Department of Pathology, Institut Universitaire du Cancer de Toulouse-Oncopole, Toulouse, France; l Centre Hospitalier Régional d'Orléans, Orléans, France; m Université Paris Centre, AP-HP, Hôpital Cochin, Paris, France; n Institut de Cancérologie de l’Ouest – ICO, site René Gauducheau, Saint-Herblain, France; o ICL Institut de Cancérologie de Lorraine, Vandoeuvre-Les-Nancy, France; p Institut Curie - Hôpital René Huguenin, Saint-Cloud, France; q Institut Gustave Roussy, Villejuif, France; r INSERM 0981, Université Paris-Saclay, France; s Centre Hospitalier Universitaire Dupuytren, Limoges, France; t Polyclinique Bordeaux Nord, Bordeaux, France; u Institut Mutualiste Montsouris, Paris, France; v Institut Bergonié, Bordeaux, France; w GINEGEPS (GINEco Group on Early Phase Studies), France; x ICO - Institut de Cancérologie de l’Ouest, site Paul Papin, Angers, France; y Hôpital Tenon, Paris, France; z Centre Georges François Leclerc, Dijon, France; aa Institut du Cancer de Montpellier - ICM Val d'Aurelle, Montpellier, France; ab Clinique Tivoli, Bordeaux, France; ac ICANS - Institut de cancérologie Strasbourg Europe, Strasbourg, France; ad Centre Antoine Lacassagne, Nice, France; ae ARCAGY-GINECO, Paris, France; af Hospices Civils de Lyon, Lyon, France

**Keywords:** Biomarker, Nucleosomes, Ovarian cancer, CA–25, KELIM

## Abstract

**Purpose:**

Circulating nucleosomes (DNA wound around histone proteins) are emerging cancer biomarkers. We investigated their association with clinical outcomes in advanced ovarian cancer.

**Patients and methods:**

Circulating levels and longitudinal kinetics of two nucleosomes (H3K27Me3 and H3K36Me3, log-scale) were assessed at baseline, during neoadjuvant chemotherapy after interval cytoreductive surgery, and at progression in patients with advanced ovarian cancer patients from the randomized phase II CHIVA trial. Nucleosomes were measured with Nu.Q® immunoassays,compared to those from 201 healthy subjects, and analyzed in relation to surgical outcomes, progression-free survival, with respect to the modeled CA–25 ELIMination rate constant K (KELIM) along with established clinical covariates.

**Results:**

H3K27Me3 and H3K36me3 nucleosome concentrations were available for 148/188 patients. Both nucleosomes were significantly higher in ovarian cancer patients compared with healthy controls and showed correlated baseline levels. Lower baseline H3K36Me3 was independently associated with complete interval cytoreduction surgery and progression-free survival, complementary to CA–25 KELIM. Longitudinal changes in nucleosome levels showed a decrease during neoadjuvant chemotherapy, but were not associated with radiological response, completeness of interval cytoreductive surgery or progression-free survival.

**Conclusion:**

Baseline H3K36Me3 and H3K27Me3 levels may represent non-invasive biomarkers in advanced ovarian cancer and warrant further evaluation for their potential clinical utility.

## Highlights

• Nucleosomes are potential diagnostic and prognostic biomarkers for ovarian cancer.
• Baseline nucleosomes did not correlate with CA–25 in advanced ovarian cancer.
• Modeled kinetics of circulating nucleosomes were not prognostic in ovarian cancer.
• Modeled kinetics of CA–25 appear to be complementary to baseline circulating nucleosomes levels.

## Introduction

Biomarkers have the potential to play a key role in the treatment of cancer by enabling early detection of cancers, predicting prognosis, and guiding treatment to allow more personalized and optimized cancer care [1,2]. Circulating tumor biomarkers may offer advantages over solid biomarkers by being less invasive and facilitating repeated measurements.

Nucleosomes are cell-free DNA comprising small fragments of chromosomes wrapped around a histone octamer core [3,4]. Epigenetic alterations, modification of chromatin without changing the DNA sequence itself, such as DNA methylation and histone post-translational modifications, may regulate gene expression and play an important role during carcinogenesis [1,2]. Nucleosomes can acquire many post-translational modifications, and different H3K27 methylation patterns were demonstrated in tumor tissue vs normal tissue [5]. Tri-methylation (Me3) of histone H3 at lysine 27 (H3K27; H3K27Me3) correlates to the inhibition of transcription, cell-cycle progression, and deregulation of cell proliferation [2,6,7].

Nucleosomes are emerging as a molecular biomarker of interest in cancer [2]. They are released into the bloodstream via various mechanisms, especially during cell death [8]. Indeed, higher levels of nucleosomes can be observed in blood samples from patients with cancer compared to those from healthy individuals [9], and may aid diagnosis. Histone methylation in nucleosomes was reported to correlate with disease stage in bladder cancer [10] and prognosis in renal cell carcinoma [11] or in breast cancer [12]. H3K27Me3 has also been observed in late-stage epithelial ovarian cancer [13], where it is thought to promote tumor vascularization and tumor cell migration. Nucleosomes may also represent a useful approach for therapeutic monitoring since they represent a biomarker for molecular residual disease [14], and their short plasma half-life can aid the detection of any changes in response to treatment [15]. In addition, the assessment of nucleosomes may provide synergistic information with other circulating cancer biomarkers [9,14].

Histone post-translational modifications (PTMs), a key epigenetic mechanism, play a central role in chromatin organization and regulation of gene expression. Aberrant histone PTM patterns have been implicated in a wide range of diseases, including cancer. Among these modifications, trimethylation of lysine 27 on histone H3 (H3K27Me3) is a well-characterized repressive epigenetic mark involved in transcriptional silencing, cell cycle regulation, and tumorigenesis. Its enzymatic regulation is driven by EZH2 (Enhancer of Zeste Homolog 2), the catalytic subunit of the Polycomb Repressive Complex 2 (PRC2), which catalyzes H3K27 trimethylation [16]. EZH2 dysregulation, through overexpression or mutation, has been frequently reported in multiple cancer types, including ovarian cancer, and is often associated with advanced tumor stages and poor prognosis [13], making it a relevant therapeutic target [17].

In contrast, H3K36Me3 is generally associated with transcriptional activation and is involved in DNA damage repair and maintenance genomic stability. It is catalyzed by the histone methyltransferase SET-domain-containing 2 (SETD2), a known tumor suppressor gene frequently altered in cancer [18]. Loss-of-function mutations in SETD2 and reduced H3K36Me3 levels have been described in several malignancies and are associated with tumor progression and genomic instability [19].

We therefore selected H3K27Me3 and H3K36Me3 as representative epigenetic marks with opposite transcriptional functions but both strongly implicated in tumor biology. Their complementary roles in oncogenic and tumor suppressor pathways provide a strong biological rationale for their evaluation as circulating biomarkers in cancer.

Cancer antigen–25 (CA–25) is a blood biomarker that has been extensively studied in patients with ovarian cancer, and that is recommended as a potential approach to monitor response to chemotherapy [20]. Mathematical modeling of circulating biomarkers is a modern and promising approach that enables the dynamic analysis of the longitudinal kinetics of tumor biomarkers during treatment [21]. Modeled CA–25 ELIMination rate constant K (KELIM) is a validated early marker of intrinsic tumor chemosensitivity in advanced epithelial ovarian carcinoma treated in first-line setting [20]. CA–25 KELIM has been reported to provide reproducible and independent prognostic value regarding progression-free survival (PFS) and overall survival in patients with ovarian cancer [[22], [23], [24]], giving insight into the likelihood of successful treatment. It is possible that, similar to CA–25, other tumor biomarkers, such as nucleosomes, may also be suitable for mathematic modeling.

CHIVA was a randomized phase II trial assessing the effect of neoadjuvant nintedanib and platinum-based chemotherapy before interval cytoreductive surgery on PFS in patients with newly epithelial ovarian cancer [25]. The utility of modeled CA–25 KELIM during neoadjuvant chemotherapy has previously been demonstrated in the CHIVA trial [24], making it a suitable study to also assess the modeled kinetics of other circulating biomarkers as well as potential relationships between those biomarkers and CA–25 KELIM. Given the observation of increased levels of nucleosomes with methylated H3K27 and H3K36 in several tumors [10,11,13,19,26], we performed a retrospective assessment of plasma samples from the CHIVA trial to examine if there was an association between circulating nucleosomes and clinical endpoints in patients with advanced ovarian cancer. In addition, we sought to understand the relationships between nucleosomes, CA–25 KELIM, and other potential predictors relative to the success of the first-line treatment in these patients.

## Material and methods

### Population and trial

CHIVA (NCT01583322) was a French multicenter phase II randomized trial led by the GINECO (Groupe d'Investigateurs Nationaux pour l'Étude des Cancers Ovariens et du sein) to evaluate the addition of nintedanib or placebo to neoadjuvant chemotherapy (carboplatin AUC 5–6 and paclitaxel 175 mg/m^2^ every 3 weeks), followed by adjuvant chemotherapy and maintenance treatment with nintedanib or placebo for up to 2 years in 188 patients with FIGO (Fédération Internationale de Gynécologie et d’Obstétrique) stage IIIC or IV epithelial ovarian cancer [25]. The protocol was approved by the ethic committee Comité de Protection des Personnes and the French health authorities Agence Nationale de Sécurité du Médicament (ANSM). The study was conducted in accordance with the Declaration of Helsinki ethical guidelines and all patients recruited in the study provided written informed consent.

During the study, data were collected on pathology subtype, grade, FIGO stage, treatment arm, completeness of cytoreduction score based on postoperative disease residues as judged by the surgeon (complete cytoreduction [CC] with no visible disease = CCO; incomplete with residues less than 2.5 mm = CC1; residues from 2.5 mm to 2.5 cm = CC2; residues more than 2.5 cm = CC3), tumor objective response to treatment according to RECIST 1.1 criteria, time interval in between the last platinum-cycle and the subsequent progression date, PFS and OS. The primary outcome of the CHIVA trial was PFS. The data from the trial have been reported previously [22].

Blood samples were obtained at the study screening visit, at the start of cycles 2 and 3, just before interval cytoreductive surgery, at each of the three adjuvant cycles, every three months during follow-up, and at progression (if any). CA–25 concentrations were assessed at local laboratories using standard tests.

To better understand how baseline CA–25 and nucleosomes interact differentially in epithelial ovarian cancer patients compared to cancer-free subjects, CA–25 and nucleosomes were also assayed in healthy individuals. K2-EDTA plasma samples from n = 201 healthy individuals were provided by Etablissement Français du Sang (Etude RNIPH 22–5065 NUCLUO CIRCAN avis CSE, 22–5065). Local institutional review board (CSE-HCL – IRB 00,013,204; AGORA 23–5274; N° d’ avis 23–5274) provided ethical approval for the samples.

### Quantification of circulating nucleosomes using Nu.Q®immunoassays

#### Blood sampling and conservation

Blood samples were collected at diagnosis and before each administration of neoadjuvant chemotherapy. For each patient, 25 mL of blood were collected on EDTA tubes. After homogenization by 8 to 10 slow turnovers, the tubes were immediately sent to the laboratory, centrifuged for 20 min (relative centrifugal force between 2500 and 3000 t/min (1500XG) to obtain a platelet poor plasma. Plasmas were then placed in cryotubes and frozen at −80 °C within one hour of collection. Blood samples at −80 °C and tumor blocks were centralized by the study promoter.

#### Quantification of circulating nucleosomes using Nu.Q®immunoassays

All samples were tested using Nu.Q® assays (Belgian Volition SRL, Isnes, Belgium). Five nucleosome structures were measured using Nu.Q® prototype immunoassays: H3.1, Nu.Q® H3K27Me3, Nu.Q® H3K36Me3, Nu.Q® H3K9Me3 and Nu.Q® H3K4Me2 immunoassays, according to the manufacturer’s instructions. Briefly, these sandwich immunoassays are based on magnetic bead and chemiluminescence technology, and are performed using the IDS-i10 automated immunoanalyzer system (Immunodiagnostic Systems Ltd. (IDS), Boldon, UK). An amount of 50 μL of K2-EDTA plasma is incubated with acridinium ester labeled as the anti-nucleosome antibody. Then, magnetic particle beads, coated with the corresponding monoclonal anti-histone modification capture antibody (i.e., anti-histone H3K27Me3, anti-histone H3K36Me3, anti-histone H3K9Me3, or anti-histone H3K4Me2, respectively), are added. Finally, after a wash step, trigger solutions are added, and the light emitted by the acridinium ester is measured via the luminometer system. The results are expressed in relative light unit (RLU), and the concentrations are extrapolated using the four-parameter logistic regression of a reference standard curve. All samples are analyzed in singlicate.

#### Modeling of circulating nucleosomes and of CA–25 longitudinal kinetics

**Fig. 1:**
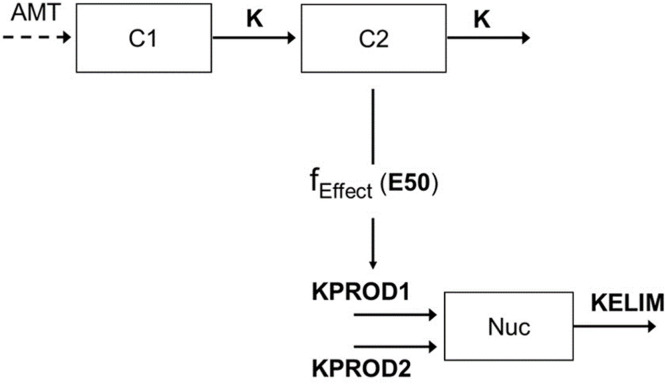
Schematic representation of the semi-mechanistic model for the modeling of nucleosome kinetics. AMT, unknown dose amount; C1, central compartment (blood); C2, peripheral compartment (transit); K, treatment kinetic rate constant (days^–^); KPROD1 and KPROD2, zero order nucleosome tumor production rate (IU.mL^–^.days^–^); E50, concentration producing 50% of the maximum effect (AU); KELIM, first order nucleosome H3K27Me3 or H3K36Me3 elimination rate (days^–^); NUc, H3K27Me3 or H3K36Me3 compartment.

The longitudinal kinetics of circulating nucleosomes and CA–25 were investigated with a population kinetic model using at least 3 available values during the first 100 days of neoadjuvant treatment. Levels of CA–25 and circulating nucleosomes were log-transformed to normalize the distribution and to avoid data being skewed to the right. The mathematical modeling of early CA–25 kinetics using a nonlinear semi-mechanistic kinetic–pharmacodynamic (K-PD) mixed-effect model was described previously [[27], [28], [29]]. The modeling of nucleosomes kinetics was based on the same model, but with 2 production rates (KPROD1 and KPROD2) (Fig. 1). Basic details about the adjustment and qualification of the model are presented in the Additional file 1 (Supplementary methods) [30].

Parameters were estimated using stochastic approximation of expectation-maximization (SAEM) algorithm and standard error (SE) was obtained by few iterations of Importance Sampling (IMP). Residual error was fixed as 1 [31]. Treatment kinetics were described by a two-compartment model comprising a central compartment (C1) receiving chemotherapy dosing and a transit compartment (C2) to describe the treatment lag-time effect. The CA–25 production inhibition induced by the treatment was expressed by an indirect effect model using an E_max_ (E50) relationship. SE of estimated parameters and goodness-of-fit plots (i.e. representations of observations relative to predictions and of normalized prediction distribution errors distribution [NPDE]) were used as the main criteria for model qualification.

#### Association between biomarkers and clinical endpoints

The association between baseline nucleosome values or modeled parameters and other potential predictors regarding tumor response rate and complete interval cytoreductive surgery (CC0) was estimated using linear regression. The statistical associations between standardized (std) KELIM and tumor response rate or interval cytoreductive surgery CC0 rate were assessed using box plots. The predictive value of std KELIM regarding the probability of obtaining a CC0 interval cytoreductive surgery was determined using a univariable/multivariable logistic regression model. Progression-free survival was estimated using univariable and multivariable Kaplan–Meier methods, log-rank, and Cox tests. The final Cox survival models were obtained using backward selections with a 5% threshold. Multivariable analyses integrated already known prognostic factors, including disease stage (IIIC vs IV); tumor histology (serous vs. others); tumor grade (well, moderately, or poorly differentiated cells); treatment arm (experimental vs standard); radiological tumor response according to RECIST criteria at the end of neoadjuvant chemotherapy, and interval cytoreductive surgery outcome for PFS analyses. Due to high levels of missing data, homologous recombination deficiency (HRD) and BRCA mutational status, which were not routinely determined at the time of inclusion in the study, were not included in analyses.

All survival analyses were implemented with a landmark time point set at 100 days after the start of neoadjuvant chemotherapy or at the surgery date, whichever occurred first. Indeed, potential biases between the prediction of clinical outcomes and the estimation of kinetics parameters had to be avoided in the case of early events, since CA–25 and nucleosome kinetics were modeled from day 0 to 100 [32]. The modeled CA–25 KELIM was standardized by the median value. The baseline nucleosome values along with the modeled kinetic parameters of interest for nucleosomes were tested as continuous and as binary covariates. The optimal cut-off was determined through the maximally selected rank statistics (MAXS). All statistical tests were implemented using a two-sided 0.05 alpha risk.

#### Statistical analysis and computing process

NONMEM 7.5.0 software (ICON Development Solutions, Ellicott City, MD, USA) was used to fit the semi-mechanistic model to CA–25 and nucleosome kinetic data. Graphical evaluation of model fits, logistic and survival analyses were performed using R software version 4.3.3© (The R Foundation, Vienna, Austria).

## Results

### Patient selection

The CHIVA trial enrolled 188 patients, of which 148 (78.7%) patients had 550 samples available for the analysis of circulating nucleosome kinetics, and 134 (71%) had 3 or more timepoints during the first 100 days for the assessment of CA–25 longitudinal kinetics. A median of 4 values of CA–25 and circulating nucleosomes measurements per patient were available. The characteristics of the patients analyzed were similar to those enrolled in the trial (Additional file 2: Supplementary Table S1).

Samples were available from 201 healthy individuals for comparison of baseline nucleosome values.

### Baseline nucleosome values

**Fig. 2:**
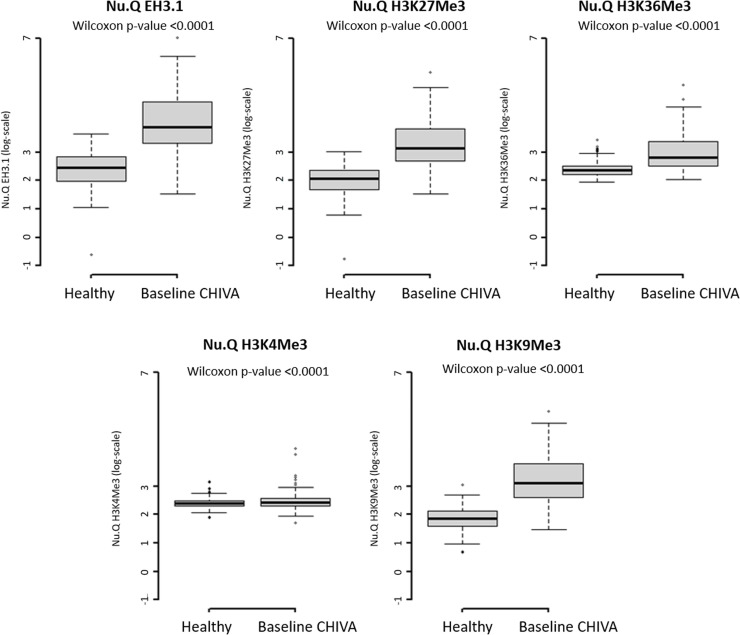
Box plots of circulating nucleosome median values in the healthy and CHIVA populations (baseline values).

In the CHIVA ovarian cancer patient population, the baseline H3K9Me3, H3K27Me3, H3K36Me3 and EH3.1 values were significantly higher than those observed in the healthy population (Fig. 2). For example, the log-scale baseline H3K27Me3 value was 3.15 (range 1.54 to 5.81; IQR 1.15) ng/mL for the CHIVA population vs 2.07 ng/mL (range −0.75 to 3.02; IQR 0.69) for the healthy population (p < 0.0001). Similarly, the log-baseline H3K36Me3 value was 2.82 ng/mL (range 2.04 to 5.36; IQR 0.83) in the CHIVA population vs 2.37 ng/mL (range 1.94 to 3.43, IQR 0.69) in the healthy population (p < 0.0001). Only H3K4Me3 levels were not significantly different between the two populations.

**Fig. 3:**
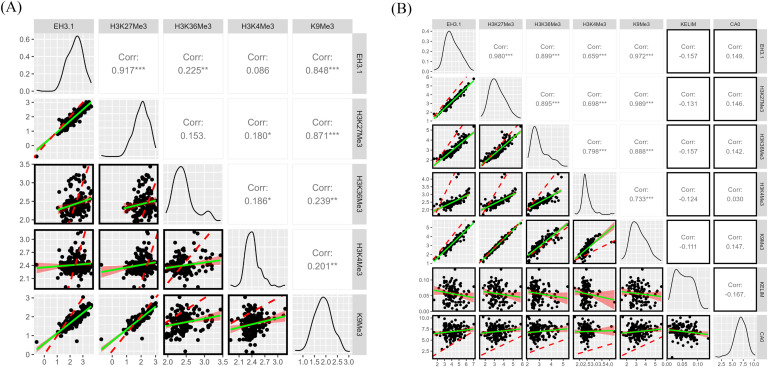
Correlation between nucleosome values in the healthy population (A) and baseline values in the CHIVA population (B). Green lines represent the linear regression, and the area around the line, the 95% confidence interval. Dashed red lines represent the identity line. Concentrations of circulating nucleosomes are represented by a log scale. Units for H3.1, H3K27, H3K36, H3K4, H3K9: ng/mL (log-scale) – panels A and B. For KELIM (panel B): day^–^; CA0: UI/mL (log-scale). * p-value between 0.01 and 0.1; ** p-value between 0.001 and 0.01; *** p-value between 0.0001 and 0.001; **** p-value < 0.0001.

There was no correlation between the values of the different nucleosomes at baseline in the healthy population (Fig. 3A). However, correlations were observed between the different nucleosome baseline values in the ovarian cancer patients enrolled in the CHIVA population (blue boxes, Fig. 3B). Therefore, further analyses focused on two nucleosomes, H3K27 and H3K36. The baseline value of nucleosomes was independent on the baseline median CA–25 values (green boxes, Fig. 3B) and the CA–25 KELIM (red boxes, Fig. 3B).

The baseline nucleosome values in the CHIVA population did not differ according to baseline patient characteristics (age and morphologic or biologic characteristics), disease characteristics (FIGO stage, histological subtype and tumor grade, Sugarbaker peritoneal carcinomatosis index) and to allocated treatment arm (Additional file 3: Supplementary Table S2).

**Fig. 4:**
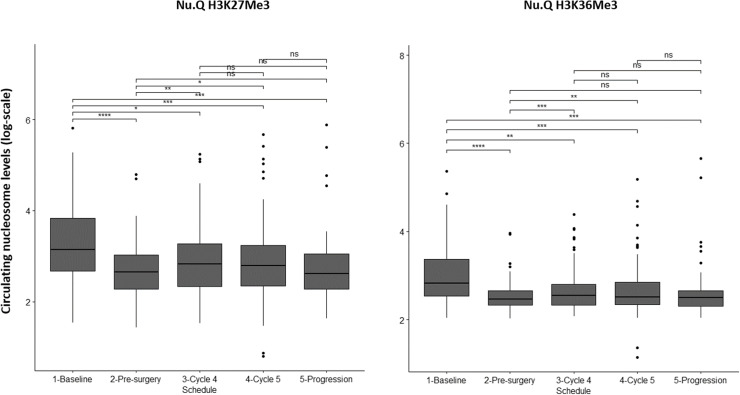
Circulating H3K27Me3 (left) and H3K36Me3 (right) nucleosome kinetics during the CHIVA therapeutic procedure. Data were analyzed by a paired-Wilcoxon test adjusted for multiple comparisons. ns = non-significant; * p-value between 0.01 and 0.1; ** p-value between 0.001 and 0.01; *** p-value between 0.0001 and 0.001; **** p-value < 0.0001.

H3K27Me3 and H3K36Me3 nucleosome kinetics changed during the neoadjuvant chemotherapy, decreasing significantly between the baseline sample and those done prior to surgery. Both levels also increased significantly after surgery (Fig. 4).

### Modeling of data and model qualification

The parameter estimates for the CA–25 kinetics model were previously reported [27]. The typical parameter estimates for the nucleosome kinetics model along with the qualification analyses from the final semi-mechanistic model are presented in the Additional file 4 (Supplementary Table S3), Additional file 5 (Supplementary Table S4), S4, Additional file 6 (Supplementary Figure S1) and Additional file 7 (Supplementary Figure S2).

Relative standard errors (RSE) of typical mean KELIM were low for H3K27Me3 (7.1%) and H3K36Me3 (1.3%). The RSE of inter-individual variability of estimates was 19.4% for H3K27Me3, but was large (140.9%) for H3K36Me3, with over shrinkage (94%). The goodness-of-fit plots suggested that individual nucleosome profiles were properly fit by the model (Additional file 6: Supplementary Figure S1 and Additional file 7: Supplementary Figure S2). Normalized prediction distribution error distributions were normally distributed for H3K27Me3 (Additional file 6: Supplementary Figure S1) and H3K36Me3 (Additional file 7: Supplementary Figure S2).

Visual predictive checks from 500 simulations suggested that most observed nucleosome values were included within 90% confidence interval boundaries of simulated H3K27Me3 and H3K36Me3 models, suggesting good predictive performance of the models. H3K27Me3 KELIM and H3K36Me3 KELIM were standardized by their median value (respectively 0.09 and 0.184), corresponding to the optimal cut-off determined by MAXS.

### Relationships between nucleosomes and tumor response rate

H3K27Me3 KELIM was higher in patients with a complete response (CR) or a partial response (PR) as the best overall response during neoadjuvant treatment, compared to those with a stable disease (SD) or a progressive disease (PD) (0.095 vs 0.090 d^–^; Wilcoxon p-value = 0.03). The baseline H3K27Me3 (Wilcoxon p-value = 0.56), the H3K27Me3 production constants (assessed by KPROD1 (Wilcoxon p-value = 0.10) and KPROD2 (Wilcoxon p-value = 0.50)), along with the percentage change in H3K27Me3 between randomization and surgery (Wilcoxon p-value = 0.73) did not differ significantly according to best overall response.

There was a non-significant trend between H3K26 KELIM and overall response rate. H3K26 KELIM was higher in patients with a best overall response of CR or PR compared those with a SD or PD (0.184 d^–^ in both groups, Wilcoxon p-value = 0.28). The baseline H3K36Me3 values (Wilcoxon p-value = 0.9) and the percentage changes in H3KMe3 between randomization and surgery were not significantly different between the patients whose best overall response was CR or PR compared to those with a SD or PD (Wilcoxon p-value = 0.48).

### Relationships between nucleosomes and the likelihood of complete interval cytoreductive surgery

Complete interval cytoreductive surgery was obtained in 74 (50.0%) patients. In univariable analysis, lower baseline H3K27Me3 (log-scale) and lower baseline H3K36Me3 (log-scale) values were both associated with a higher probability of obtaining complete cytoreduction (CC0) at interval surgery (H3K27Me3: odds ratio [OR] 0.55 [95% confidence interval [CI] 0.35–0.85], p = 0.008; H3K36Me3: OR 0.50 [95%CI 0.29–0.83], p = 0.009) (Additional file 8: Supplementary Table S5). However, no associations were observed between complete cytoreduction and pre-surgery H3K27Me3 values (OR 0.80 [95%CI 0.40–1.58], p = 0.51), pre-surgery H3K36Me3 values (OR 0.46 [95%CI 0.13–1.52], p = 0.20), or the percentage of change between randomization and surgery in H3K27Me3 (OR 1.00 [95%CI 0.99–1.02], p = 0.66) or in H3K36Me3 values (OR 1.01 [95%CI 0.98–1.04], p = 0.47). No association was observed between interval cytoreductive surgery CC0 and standardized KELIM or the production parameters (KPROD1 and KPROD2) for either H3K27Me3 or H3K36Me3 (Additional file 8: Supplementary Table S5).

In the final multivariable model, the significant covariates for obtaining an interval cytoreductive surgery CC0 were CA–25 KELIM (OR 10.54 [95%CI 3.92–33.37], p < 0.001) and baseline H3K36Me3 (log-scale) values (0.33 [95%CI 0.13–0.71], p = 0.009).

### Prognostic value of nucleosomes baseline and kinetic parameters regarding progression-free survival

#### Univariable analyses

**Fig. 5:**
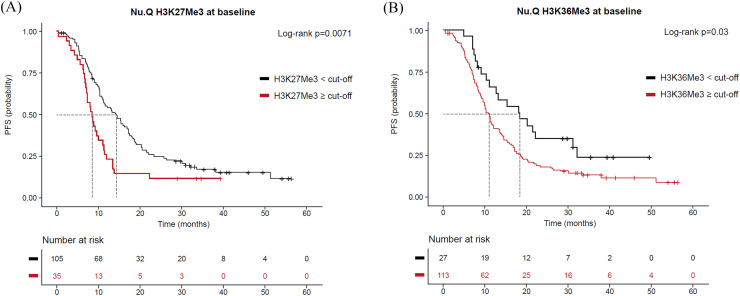
Progression-free survival according to the baseline values of H3K27Me3 nucleosomes (A) and H3K36Me3 nucleosomes (B). Survival curves were estimated using the Kaplan–Meier method, and group comparisons were performed using a two-sided log-rank test. P-values are reported accordingly.

A total of 140 patients were assessable for PFS analyses. The optimal cut-off for baseline H3K27Me3 (log-scale) values using MAXS was 3.79 ng/mL, whereas the median value was 3.15 ng/mL. Median PFS was 14.4 months (95%CI 11.2–17.2) in the patients with a baseline H3K27Me3 (log-scale) < 3.79 ng/mL, against 8.5 months (95%CI 7.2–11.4) for those with a H3K27Me3 ≥ 3.79 ng/mL (log-rank p = 0.007; Figure 5; HR 0.57 (95%CI 0.38–0.87), p = 0.008). However, neither the percentage change between randomization and surgery, nor the standardized H3K27Me3 KELIM or the production parameters (KPROD1 and KPROD2) were associated with PFS (Additional file 9: Supplementary Table S6).

The optimal cut-off for baseline H3K36Me3 (log-scale) values using MAXS was 2.45 ng/mL, whereas the median value was 2.82 ng/mL. Median PFS was 18.5 months (95%CI 12.9-not reached [NR]) for patients with a baseline H3K36Me3 level < 2.45 ng/mL vs 10.9 months (95%CI 9.6–13.5) when ≥ 2.45 ng/mL (log-rank p = 0.03; Fig. 5). Univariable Cox regression analysis indicated an association between lower baseline H3K36Me3 and longer PFS (HR 0.58 [95%CI 0.36–0.95], p = 0.032) (Additional file 9: Supplementary Table S6). No association was observed between PFS and the percentage change in H3K36Me3 between randomization and surgery (Additional file 9: Supplementary Table S6). Due to substantial shrinkage observed in the longitudinal model of H3K36Me3, derived kinetic parameters were considered insufficiently robust for further association analyses with clinical outcomes.

#### Multivariable analysis

The data from 174 patients were available for a multivariable analysis. The two factors independently associated with longer PFS were: a baseline value of H3K27Me3 lower than the optimal cut-off of 3.79 ng/mL as determined by MAXS (log-scale) (HR 0.54 [95%CI 0.33–0.90], p = 0.016), and a favorable (≥ 1.0) CA–25 KELIM score (HR 0.45 [95%CI 0.29–0.70], p < 0.001). Baseline H3K36Me3 (log-scale) was not significantly associated with PFS in multivariable analyses.

#### Bivariable analyses of baseline nucleosome values and CA–25 Kelim

**Fig. 6:**
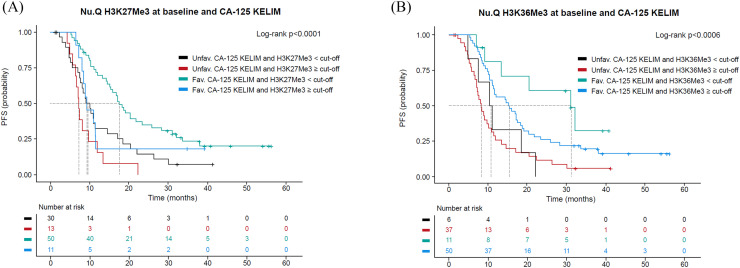
Progression-free survival according to CA–25 KELIM and baseline H3K27Me3 nucleosomes (A) or baseline H3K36Me3 nucleosomes (B). Survival curves were estimated using the Kaplan–Meier method, and group comparisons were performed using a two-sided log-rank test. P-values are reported accordingly.

When considering both CA–25 KELIM and baseline H3K27Me3 levels, we identified a good prognosis population, characterized by a favorable CA–25 KELIM (≥ 1.0) and a low baseline H3K27Me3 level (< 3.15 ng/mL). In this subgroup, the median PFS was 17.5 months [95% CI 15.3–25.9] and was significantly longer than PFS in the other groups. Patients with an unfavorable CA–25 KELIM and baseline H3K27Me3 levels ≥ cut-off had a median PFS of 7.2 months (95%CI 6.4-NR). PFS reached 9.6 months [95%CI 7.6–17.2] for those with an unfavorable CA–25 KELIM and baseline H3K27Me3 levels < cut-off, and 9.3 months (95%CI 8.5-NR) for patients with a favorable CA–25 KELIM and a baseline H3K27Me3 level ≥ cut-off (log-rank p < 0.001) (Fig. 6).

Similarly, median PFS was higher in patients with a favorable CA–25 KELIM and a baseline H3K36Me3 level < cut-off (31.1 months [95%CI 13.3-NR]) than other patients. When CA–25 KELIM was unfavorable and baseline H3K36 levels ≥ cut-off, median PFS was 8.2 months (95%CI 7.1-NR). It reached 10.7 months (95%CI 7.5-NR) in patients with an unfavorable CA–25 KELIM and baseline H3K36Me3 < cut-off, and 15.4 months (95%CI 11;4–19;1) in case of favorable CA–25 KELIM and baseline H3K36Me3 ≥ cut-off (Log-rank p < 0.001) (Fig. 6).

## Discussion

In our study, higher baseline levels of nucleosomes were associated with poorer prognosis in patients with advanced ovarian cancer, potentially reflecting more aggressive tumor biology. Baseline H3K36Me3 levels were also independent predictors of completeness of cytoreduction at interval surgery. In addition, our study confirmed that mathematical modeling of H3K27Me3 longitudinal kinetics was feasible, although the modeled kinetic parameters did not provide additional prognostic value beyond baseline measurements.

We also observed higher nucleosome levels in patients with advanced ovarian cancer compared with healthy controls. Interestingly, baseline nucleosome levels were not correlated with CA–25 values, suggesting that these biomarkers may provide complementary information to CA–25. However, dedicated studies including early-stage disease and benign controls are required to determine their potential diagnostic utility.

Circulating cell-free DNA may represent a useful approach for cancer biomarker development across several tumor types [33]. Similar to our findings, elevated circulating H3K27Me3 nucleosome concentrations have been reported in patients with non-small cell lung cancer compared with healthy controls [14]. Levels of methylated H3K27 were also reported to be higher in bladder cancer compared to normal urothelium [10]. However, these observations contrast with a study in which H3K27Me3 levels were higher in benign renal tissue than in recurrent renal cell carcinomas [11]. These discrepancies suggest that the biological and clinical significance of H3K27 methylation may differ according to tumor type. For example, in ovarian cancer, H3K27 hypermethylation has been associated with late-stage disease, promotion of tumor vascularization, and cell migration, whereas in diffused midline glioma, it is thought to contribute to tumor suppressor gene silencing [13].

Caution is warranted when interpreting longitudinal changes in circulating nucleosome levels, as they may be influenced not only by tumor-related processes, but also by systemic inflammation, treatment-induced cell death and surgical tissue injury [2]. In particular the postoperative increase observed in our study may partly reflect surgery-related tissue damage and inflammatory responses, consistent with the well-documented transient rise of other circulating biomarkers after cytoreductive surgery [34], including total cell-free DNA [35] and CA–25 [36]. This constitutes an important limitation for postoperative nucleosome monitoring, as surgery-induced nucleosome release may mask residual disease signals during a critical window for minimal residual disease assessment. Importantly, our prognostic analyses were performed in the neoadjuvant setting using baseline samples collected before interval cytoreductive surgery, thereby limiting potential confounding effects of surgical trauma and postoperative inflammatory responses on circulating nucleosome levels. These findings nonetheless highlight the importance of considering sampling timing and clinical context when interpreting circulating nucleosome kinetics, and suggest that postoperative monitoring would require approaches capable of distinguishing tumor-derived from injury-derived kinetics.

Although increased tissue methylated H3K27 nucleosome levels have been associated with more advanced tumor stages in prostate and bladder cancers [5,10], we did not observe a correlation between circulating methylated nucleosomes and FIGO stage (IIIC vs IV) in our study. This may reflect the relatively homogeneous population included in our study, restricted to high-risk unresectable FIGO IIIC-IV ovarian cancer. Therefore, associations between circulating methylated nucleosomes and tumor stage cannot be excluded. Discussion around tumor burden and patterns of peritoneal dissemination may also be of interest.

The two nucleosome markers appeared to reflect distinct biological and clinical dimensions of ovarian cancer. Baseline H3K36Me3 levels were associated with the likelihood of incomplete cytoreductive surgery, whereas H3K27Me3 levels were associated with progression-free survival. These differences are consistent with the known epigenetic functions of these histone modifications, with H3K36Me3 linked to active transcription and cellular proliferation, and H3K27Me3 to transcriptional repression and epigenetic silencing. Although these findings support a degree of biological complementarity between the two markers, simultaneous inclusion of both biomarkers did not improve prognostic performance in our cohort. This suggests that their clinical relevance may be endpoint-specific, with each marker capturing distinct aspects of tumor biology. The correlation that we observed between lower values of H3K27Me3 and H3K36Me3 and improved tumor prognosis was consistent with studies in patients with other cancers. In patients with diffuse large B-cell lymphoma, higher levels of expression of tissue H3K27Me3 were associated with poor response to treatment, worse remission rates and shorter overall survival [37]. In advanced non-small-cell lung cancer, high levels of baseline plasma nucleosomes, and low decrease during first-line chemotherapy was also associated with poorer response to therapy and disease progression [38]. In contrast, a study in patients with renal cell carcinoma reported that lower levels of tissue H3K27Me3 and H3K27Me1 were associated with shorter PFS in univariable analysis. Moreover, nucleosome histone modifications were not associated with disease recurrence or survival in non-muscle invasive bladder cancer [10]. These observations suggest potential differences in the prognostic value of H3K27 between cancers and between circulating vs tissue levels. These observations suggest potential differences in the prognostic value of histone modifications between cancer types and between circulating versus tissue levels. Beyond tumor-specific epigenetic landscapes, the mode of tumor dissemination may also contribute to These observations suggest potential differences in the prognostic value of histone modifications between cancer types and between circulating versus tissue levels. Beyond tumor-specific epigenetic landscapes, the mode of tumor dissemination may also contribute to these discrepancies. Unlike many solid tumors that spread predominantly hematogenously, advanced ovarian cancer is characterized by peritoneal carcinomatosis, in which epigenetic dynamics may play a key role in tumor adaptation and dissemination [39]. Nucleosomes may reach the bloodstream indirectly, through lymphatic drainage or peritoneal–vascular exchanges, rather than direct tumor–vessel contact, potentially altering their epigenetic composition and producing circulating signatures that diverge from those observed in hematogenously spreading tumors.

However, based on our study, circulating nucleosomes may have some potential utility for predicting prognosis in advanced ovarian cancer.

CA–25 KELIM was previously shown to be associated with the tumor chemosensitivity, the likelihood of complete cytoreduction at interval surgery, PFS, and subsequent platinum-free intervals in patients with ovarian cancer in the CHIVA trial [24]. There is a need for biomarkers that may complement the use of CA–25 in the diagnosis and management of ovarian cancer. In our analysis, baseline nucleosomes did not appear to correlate with CA–25 in patients newly diagnosed with advanced epithelial ovarian cancer. However, CA–25 KELIM appeared to be complementary with baseline H3K27Me3 for prognosis, and with H3K36Me3 for predicting complete cytoreduction at interval surgery. Importantly, while combining CA–25 KELIM with either baseline H3K27Me3 or H3K36Me3 improved prognostic stratification, simultaneously including both nucleosome markers did not further enhance prognostic performance, suggesting either overlapping biological information or endpoint-specific clinical relevance — H3K27Me3 appearing more relevant to survival outcomes and H3K36Me3 to surgical resectability.

Molecular profiling and circulating nucleosome measurements could be two complementary approaches. Circulating tumor DNA (ctDNA) analysis by Next Generation Sequencing allows to detect targetable mutations. Thus, tumor genetic testing has become an important parameter in the choice of therapies at diagnosis but also to detect Minimal Residual Disease (MRD). However, clinical interpretation of ctDNA negative remains challenging. Indeed, sensitivity is limited not only by low mutant allele frequency, but also subject to variation depending on the technics used. These false negative results may be linked to biological and technological limitations. Grolleau et al. have described for non-small cell lung cancer, that 27% of ctDNA negative patients during treatment were H3K27Me3-nucleosomes positive [14]. This could illustrate a progression of the disease in the absence of acquisition of known somatic mutation or mutation detected by the NGS panel used. In addition, Piecyk et al. have demonstrated that H3K27Me3-nucleosome could predict survival regardless of the mutational status [40].

Our study did not assess the correlation of nucleosomes with other plasma biomarkers for ovarian cancer, such as human epididymis protein (HE4), that may be used to aid diagnosis [20,41,42]. HE4 could provide complementary value to nucleosomes for the diagnosis and determination of prognosis in ovarian cancer, and such an approach would warrant investigation in future studies.

Several limitations should be considered. Firstly, the data were derived from a small number of patients, which would have reduced the power of statistical analyses to detect associations. Nucleosomes have a short half-life (∼2 hour in plasma), meaning that the exploration of kinetic analyses may be subject to time-dependent variability related to sampling conditions. It should also be noted that current nucleosome assays have their limitations, requiring further optimization and standardization of preanalytical steps, as well as analytical procedures.

Additionally, the longitudinal kinetic modeling of H3K36Me3 revealed substantial inter-individual variability and model overshrinkage, precluding robust estimation of individual kinetic parameters and, consequently, any meaningful assessment of their prognostic value. As this analysis relies on a single cohort, it remains unknown whether these modeling limitations would persist in an independent dataset, and whether H3K36Me3 kinetics during treatment carries prognostic information warrants investigation in future studies.

Then, the use of maximally selected cutpoints may increase the risk of overfitting in small exploratory cohorts despite being a widely used method for survival data. Therefore, these cutpoint-based analyses should be interpreted as hypothesis-generating and require independent external validation. Although continuous modelling approaches may offer additional statistical information, this study primarily focused on developing clinically meaningful risk stratification in a translational context. The optimal validation strategy would follow a two-step approach: first, the pre-specified thresholds identified here (H3K27Me3 ≥ 3.79 ng/mL and H3K36Me3 ≥ 2.45 ng/mL) should be applied as fixed, pre-defined cut-points in an independent cohort of newly diagnosed ovarian cancer patients treated with neoadjuvant chemotherapy, thereby providing a genuine test of their prognostic value. Complementarily, re-applying MAXS within the validation cohort and examining whether the resulting thresholds converge toward similar values would further support the biological robustness of these cut-points.

Although BRCA mutational and HRD status are now recommended at diagnosis for patients with high-grade disease [20], this was not standard practice in the first-line setting when the CHIVA trial started. Consequently, this information was not available for ∼45% of the patients, limiting the interpretability of any association with circulating nucleosomes. As HRD status may influence tumor biology and therefore nucleosome release dynamics, we cannot exclude that it acts as a confounding factor in the prognostic value attributed to nucleosome levels observed here. Future prospective studies incorporating systematic BRCA and HRD testing will therefore be essential to determine whether nucleosome kinetics retain independent prognostic value across molecular subgroups.

Further studies in larger cohorts, together with assay refinement and external validation, are required before nucleosomes can be widely adopted as biomarkers in ovarian cancer.

## Conclusion

This exploratory post hoc analysis supports the potential role of circulating nucleosomes as baseline levels as complementary prognostic biomarkers to CA–25 in ovarian cancer. While longitudinal kinetic modeling of CA–25 (KELIM) proved both feasible and prognostically informative, kinetic modeling of H3K27me3 was feasible but did not add prognostic value beyond baseline levels, and that of H3K36Me3 was limited by substantial inter-individual variability and model overshrinkage, precluding conclusions on its prognostic relevance. Their distinct longitudinal patterns may reflect different aspects of tumor biology and disease evolution. Prospective validation in larger cohorts is warranted, together with integrated studies assessing their interplay with other biomarkers, including circulating tumor DNA, for diagnostic and prognostic applications.

## Disclosure

PCorbaux: Novartis, Pfizer, Daiichi Sankyo, AstraZeneca.

IRC: Adaptimmune, Agenus, Amgen, Abbvie, AstraZeneca, BioNtech, LoxoLilly, BMS, Clovis, Corcept, Daiichi Sankyo, Deciphera, Eisai, EQRx, GSK, Genmab, Gilead, MacroGenics, Merck Serono, Mersana, Novartis, PVM pharma, Onxeo, Roche, Sutro Biopharma, Scorpion, Pharmamar.

FJ: GSK, AstraZeneca, Clovis Oncology, Seagen, Janssen, Bayer, MSD, Ipsen, Astellas; Amgen, GSK, Bayer, Eisai, MSD, IPSEN.

JA: Astra Zeneca, GSK, Pfizer, Eisai, MSD, Janssen, Novartis.

DBR: Daiichi Sankyo, Lilly.

ND: Pfizer, Seagen, Roche, Daiichi Sankyo, AstraZeneca, Lilly.

AL: Medscape, GLG, Servier; AstraZeneca, Oseimmuno.

CLebreton: GSK, Abbvie, EISAI, AstraZeneca, MSD, Clovis Oncology.

JK: GSK, AstraZeneca, Eisai, MSD, Abbvie.

PF: AstraZeneca, EISAI, Gilead, GSK, MSD, Novartis EPL: GSK, Incyte, Agenus.

BY: MSD, AstraZeneca, GSK-TESARO, BAYER, Roche-Genentech, ECS Progastrin, Novartis, LEK, Amgen, Clovis Oncology and Merck Serono.

The other authors report no conflicts of interest in this work.

## Funding

AstraZeneca.

## CRediT authorship contribution statement

**Pauline Corbaux:** Writing – original draft, Formal analysis. **Olivier Colomban:** Writing – review & editing, Formal analysis. **Gaelle Lescuyer:** Writing – review & editing, Formal analysis. **Isabelle Ray-Coquard:** Writing – review & editing, Investigation. **Gaëtan De Rauglaudre:** Writing – review & editing, Investigation. **Florence Joly:** Writing – review & editing, Investigation. **Cyril Abdeddaim:** Writing – review & editing, Investigation. **Pierre Combe:** Writing – review & editing, Investigation. **Cyriac Blonz:** Writing – review & editing, Investigation. **Guillaume Bataillon:** Writing – review & editing, Investigation. **Jérôme Meunier:** Writing – review & editing, Investigation. **Jérôme Alexandre:** Writing – review & editing, Investigation. **Dominique Berton:** Writing – review & editing, Investigation. **Marie-Christine Kaminsky:** Writing – review & editing, Investigation. **Diana Bello Roufai:** Writing – review & editing, Investigation. **Alexandra Leary:** Writing – review & editing, Investigation. **Laurence Venat:** Writing – review & editing, Investigation. **Nadine Dohollou:** Writing – review & editing, Investigation. **Christophe Louvet:** Writing – review & editing, Investigation. **Coriolan Lebreton:** Writing – review & editing, Investigation. **Sophie Abadie-Lacourtoisie:** Writing – review & editing, Investigation. **Jean-Pierre Lotz:** Writing – review & editing, Investigation. **Laure Favier:** Writing – review & editing, Investigation. **Michel Fabbro:** Writing – review & editing, Investigation. **Nathalie Bonichon-Lamichhane:** Writing – review & editing, Investigation. **Jean-Emmanuel Kurtz:** Writing – review & editing, Investigation. **Philippe Follana:** Writing – review & editing, Investigation. **Eric Pujade-Lauraine:** Writing – review & editing, Investigation. **Lea Payen:** Writing – review & editing, Writing – original draft, Supervision, Methodology, Formal analysis. **Benoit You:** Writing – review & editing, Writing – original draft, Supervision, Methodology, Investigation, Conceptualization.

## Declaration of competing interest

The authors declare the following financial interests/personal relationships which may be considered as potential competing interests:

GINECO reports equipment, drugs, or supplies was provided by AstraZeneca. Pauline Corbaux reports a relationship with Novartis that includes: travel reimbursement. Pauline Corbaux reports a relationship with Pfizer Inc that includes: travel reimbursement. Pauline Corbaux reports a relationship with Daiichi Sankyo Inc that includes: travel reimbursement. Pauline Corbaux reports a relationship with AstraZeneca that includes: travel reimbursement. Pauline Corbaux reports a relationship with GSK that includes: consulting or advisory and speaking and lecture fees. Isabelle Ray-Coquard reports a relationship with Adaptimmune LLC that includes:. Isabelle Ray-Coquard reports a relationship with Amgen Inc that includes:. Isabelle Ray-Coquard reports a relationship with AbbVie Inc that includes:. Isabelle Ray-Coquard reports a relationship with AstraZeneca that includes:. Isabelle Ray-Coquard reports a relationship with BioNTech SE that includes:. Isabelle Ray-Coquard reports a relationship with Loxo Oncology Inc that includes:. Isabelle Ray-Coquard reports a relationship with Bristol Myers Squibb Co that includes:. Isabelle Ray-Coquard reports a relationship with Clovis Oncology Inc that includes:. Isabelle Ray-Coquard reports a relationship with Corcept Therapeutics Inc that includes:. Isabelle Ray-Coquard reports a relationship with Daiichi Sankyo Inc that includes:. Isabelle Ray-Coquard reports a relationship with Deciphera Pharmaceuticals that includes:. Isabelle Ray-Coquard reports a relationship with Eisai Inc that includes:. Isabelle Ray-Coquard reports a relationship with EQRx, Inc. that includes:. Isabelle Ray-Coquard reports a relationship with GSK that includes:. Isabelle Ray-Coquard reports a relationship with Genmab that includes:. Isabelle Ray-Coquard reports a relationship with Gilead Sciences Inc that includes:. Isabelle Ray-Coquard reports a relationship with MacoGenics that includes:. Isabelle Ray-Coquard reports a relationship with Merck Serono that includes:. Isabelle Ray-Coquard reports a relationship with Mersana Therapeutics Inc that includes:. Isabelle Ray-Coquard reports a relationship with Novartis that includes:. Isabelle Ray-Coquard reports a relationship with Roche SAS that includes:. Isabelle Ray-Coquard reports a relationship with Sutro Biopharma Inc that includes:. Isabelle Ray-Coquard reports a relationship with PharmaMar USA Inc that includes:. Florence Joly reports a relationship with GSK that includes: consulting or advisory. Florence Joly reports a relationship with AstraZeneca that includes: consulting or advisory. Florence Joly reports a relationship with Clovis Oncology Inc that includes: consulting or advisory. Florence Joly reports a relationship with Seagen Inc that includes: consulting or advisory. Florence Joly reports a relationship with Eisai Inc that includes:. Florence Joly reports a relationship with Janssen Pharmaceuticals Inc that includes: consulting or advisory. Florence Joly reports a relationship with Bayer Corporation that includes: consulting or advisory. Florence Joly reports a relationship with Merck & Co Inc that includes: consulting or advisory. Florence Joly reports a relationship with Ipsen Pharma SAS that includes: consulting or advisory. Florence Joly reports a relationship with Amgen Inc that includes: speaking and lecture fees. Florence Joly reports a relationship with Astellas Pharma US Inc that includes: consulting or advisory. Florence Joly reports a relationship with GSK that includes: speaking and lecture fees. Florence Joly reports a relationship with Bayer Corporation that includes: speaking and lecture fees. Florence Joly reports a relationship with Eisai Inc that includes: speaking and lecture fees. Florence Joly reports a relationship with Merck & Co Inc that includes: speaking and lecture fees. Florence Joly reports a relationship with GSK that includes: travel reimbursement. Florence Joly reports a relationship with Merck & Co Inc that includes: travel reimbursement. Florence Joly reports a relationship with Eisai Inc that includes: travel reimbursement. Florence Joly reports a relationship with Ipsen SA that includes: travel reimbursement. Jerome Alexandre reports a relationship with AstraZeneca that includes:. Jerome Alexandre reports a relationship with GSK that includes:. Jerome Alexandre reports a relationship with Pfizer that includes:. Jerome Alexandre reports a relationship with Eisai Inc that includes:. Jerome Alexandre reports a relationship with Merck & Co Inc that includes:. Jerome Alexandre reports a relationship with Janssen Pharmaceuticals Inc that includes:. Jerome Alexandre reports a relationship with Novartis that includes:. Nadine Dohollou reports a relationship with Pfizer Inc that includes: consulting or advisory. Nadine Dohollou reports a relationship with Seagen Inc that includes: speaking and lecture fees. Nadine Dohollou reports a relationship with Roche that includes: consulting or advisory. Nadine Dohollou reports a relationship with Daiichi Sankyo Inc that includes:. Nadine Dohollou reports a relationship with AstraZeneca Pharmaceuticals LP that includes:. Nadine Dohollou reports a relationship with Lilly France that includes:. Alexandra Leary reports a relationship with Medscape LLC that includes:. Alexandra Leary reports a relationship with Servier Monde that includes:. Alexandra Leary reports a relationship with AstraZeneca that includes:. Alexandra Leary reports a relationship with OSE Immunotherapeutics SA that includes:. Coriolan Lebreton reports a relationship with GSK that includes: consulting or advisory and travel reimbursement. Coriolan Lebreton reports a relationship with AbbVie Inc that includes: consulting or advisory. Coriolan Lebreton reports a relationship with Eisai Inc that includes: consulting or advisory. Coriolan Lebreton reports a relationship with AstraZeneca that includes: consulting or advisory. Coriolan Lebreton reports a relationship with Merck & Co Inc that includes: consulting or advisory and travel reimbursement. Coriolan Lebreton reports a relationship with Clovis Oncology that includes: consulting or advisory. Jean-Emmanuel Kurtz reports a relationship with GSK that includes: consulting or advisory. Jean-Emmanuel Kurtz reports a relationship with AstraZeneca Pharmaceuticals LP that includes: consulting or advisory. Jean-Emmanuel Kurtz reports a relationship with AbbVie Inc that includes: consulting or advisory. Jean-Emmanuel Kurtz reports a relationship with Eisai Inc that includes: consulting or advisory. Jean-Emmanuel Kurtz reports a relationship with MSD France SAS that includes: consulting or advisory. Philippe Follana reports a relationship with AstraZeneca Pharmaceuticals LP that includes: consulting or advisory. Philippe Follana reports a relationship with Eisai Inc that includes: speaking and lecture fees. Philippe Follana reports a relationship with Gilead Sciences Inc that includes: travel reimbursement. Philippe Follana reports a relationship with GSK that includes: speaking and lecture fees. Philippe Follana reports a relationship with MSD France SAS that includes: speaking and lecture fees. Philippe Follana reports a relationship with Novartis Pharma SAS that includes: consulting or advisory. Eric Pujade-Lauraine reports a relationship with Incyte Corporation that includes: consulting or advisory. Eric Pujade-Lauraine reports a relationship with Agenus Inc that includes: consulting or advisory. Eric Pujade-Lauraine reports a relationship with GSK that includes:. Benoit You reports a relationship with MSD that includes: consulting or advisory. Benoit You reports a relationship with AstraZeneca that includes: consulting or advisory. Benoit You reports a relationship with GSK that includes: consulting or advisory. Benoit You reports a relationship with Bayer Corporation that includes: consulting or advisory. Benoit You reports a relationship with Roche-Genentech that includes: consulting or advisory. Benoit You reports a relationship with ECS Progastrin that includes: consulting or advisory. Benoit You reports a relationship with Novartis Pharma SAS that includes: consulting or advisory. Benoit You reports a relationship with LEK that includes: consulting or advisory. Benoit You reports a relationship with Amgen Inc that includes: consulting or advisory. Benoit You reports a relationship with Clovis Oncology Inc that includes: consulting or advisory. Benoit You reports a relationship with Merck Serono SA that includes: consulting or advisory. If there are other authors, they declare that they have no known competing financial interests or personal relationships that could have appeared to influence the work reported in this paper.

## Appendix Supplementary materials

Image, application 1
