# Supplementary material for: Baseline value and longitudinal kinetics of circulating nucleosomes during neo-adjuvant chemotherapy in newly diagnosed ovarian cancer: Results from a GINECO/GINEGEPS study of the randomized phase II CHIVA trial
Source: Transl Oncol. 2026 Jul 1;71:102896. doi: 10.1016/j.tranon.2026.102896 (PMC13342990; doi:10.1016/j.tranon.2026.102896)
Supplement: Supplementary file 1 [file mmc1.docx]

# **SUPPLEMENTARY MATERIAL**

**Contents:**

**Supplementary methods**

**Supplementary Table S1:** Characteristics of the analysis populations

**Supplementary Table S2.** Baseline nucleosome values according to disease characteristics and treatment arm

**Supplementary Table S3:** Typical parameter estimates from the final semi-mechanistic model for circulating H3K27Me3 nucleosomes

**Supplementary Table S4:** Typical parameter estimates from the final semi-mechanistic model for circulating H3K36Me3 nucleosomes

**Supplementary Table S5:** Univariable logistic prediction of the probability of obtaining a complete interval cytoreductive surgery

**Supplementary Table S6:** Cox regression models of progression-free survival.

**Supplementary Figure S1:** Goodness-of-fit plots for H3K27 modeling

**Supplementary Figure S2:** Goodness-of-fit plots for H3K36 modeling

### Supplementary methods

*Basic Population modeling*

CA-125 data are described as follows:

$y_{ij}=f\left( X_{ij},P_{i} \right)+\varepsilon_{ij}$

Where $y_{ij}$corresponds to the j^th^ observation of the i^th^ individual, $f\left( \ldots\right)$is the structural model described by the variables $X_{ij}$ (time and dose), $P_{i}$is the vector of individual parameter for the i^th^ individual and $\varepsilon_{ij}$is the residual error defined as the difference between observed data $y_{ij}$and individual predictions $\hat{y_{ij}}. \varepsilon$ is assumed to be normally distributed with zero mean and variance to be estimated**.**

The individual parameters $P_{i}$are distributed around their typical values in the population $\theta$(fixed effect or population parameter). Deviations from this typical value were expressed according to a log-normal distribution (random effect):

$P_{i}=\theta\times e^{\eta_{i}}$

Where $\theta$is the typical value of the parameter P (mean in the population)-the random effect $\eta_{i}$ describes the inter-individual variability (IIV) which is the difference between the population value and the i^th^ individual value. $\eta_{i}$values are expected to be a normal distribution with a 0 mean and $\omega^{2}$variance**.**

*Structural model and model qualification*

Treatment kinetics were described by a two-compartment model: central compartment (C1) receiving chemotherapy dosing (doses set to 1) and a transit compartment (C2) to describe the treatment lag-time effect. The CA-125 production inhibition induced by the treatment is expressed by an indirect effect model using an E_max_ (E50) relationship.

Where K is the treatment kinetic rate constant (days^-1^); KPROD is the CA-125 tumor production rate (IU.mL^-1^days^-1^); E50 is the concentration producing 50% of the maximum effect (AU); KELIM is the CA-125 elimination rate (days^-1^); and CA0 is the estimated CA-125 at time = 0 (IU.mL^-1^).Standard errors of estimated parameters and goodness-of-fit plots (i.e. plots of observations vs predictions and of the distribution of normalized prediction errors [NPDE]) were used as major criteria. Moreover, 500 replicates of all individual CA-125 decline profiles were simulated using the final population model to perform a visual predictive check (VPC): the 10^th^, 50^th^, and 90^th^ percentiles of the observed CA-125 values were compared with the 95% CI computed from the 500 simulated replicates. The observed and simulated median vs time profiles were visually compared.

### Supplementary Table S1. Characteristics of the analysis populations

|  | **Global population  (N = 185*)** | **Population for nucleosome analyses  (N = 148)** |
| --- | --- | --- |
| **Continuous covariates, median (min-max)** | | |
| Age (years) | 64 [31-79] | 64.5 [31.0-79.0] |
| Height (cm) | 161 [148-180] | 160.0 [148.0-180.0] |
| Weight (kg) | 64 [40-103] | 64.0 [40.0-103.0] |
| **Categorical covariates, n (%)** | | |
| FIGO stage at diagnosis |  |  |
| Stage IIIc | 144 (77.8) | 116 (78.4) |
| Stage IV | 41 (22.2) | 32 (21.6) |
| Histology |  |  |
| Serous/papillary | 162 (87.6) | 131 (88.5) |
| Others | 23 (12.4) | 17 (11.5) |
| Histological grade |  |  |
| Grade 1 | 5 (2.7) | 4 (2.7) |
| Grade 2 | 13 (7.0) | 10 (6.8) |
| Grade 3 | 133 (71.9) | 104 (70.3) |
| Missing data | 34 (18.4) | 30 (20.3) |
| Treatment Arm |  |  |
| Nintedanib | 124 (67.0) | 102 (68.9) |
| Placebo | 61 (33.0) | 46 (31.1) |
| GCIG CA-125 Response |  |  |
| Unfavorable | 41 (22.2) | 32 (21.6) |
| Favorable | 93 (50.3) | 74 (50.0) |
| Missing data | 51 (27.6) | 42 (28.4) |
| Completeness of the IDS |  |  |
| Complete | 92 (49.7) | 74 (50.0) |
| Incomplete | 93 (50.3) | 71 (48.0) |
| Missing data | 0 | 3 (2.0) |
| BRCA 1 or 2 mutation |  |  |
| Yes | 27 (14.6) | 21 (14.2) |
| No | 102 (55.1) | 81 (54.7) |
| Missing data | 56 (30.3) | 46 (31.1) |
| HRD Status |  |  |
| BRCA mutation | 27 (14.6) | 21 (14.2) |
| BRCAwt/HRD | 29 (15.7) | 27 (18.2) |
| BRCAwt/HRP | 47 (25.4) | 34 (23.0) |
| Missing data | 82 (44.3) | 66 (44.6) |

*3 of the 188 patients in the CHIVA analysis population without available data

HRD, homologous recombination deficiency; HRP, homologous recombination proficiency; IDS, interval debulking surgery; wt, wild-type

| **Supplementary Table S2.** Baseline nucleosome values according to disease characteristics and treatment arm | | | | | | |
| --- | --- | --- | --- | --- | --- | --- |
|  |  | **H3K27**  **(ng/mL, log-scale)** | | | **H3K36**  **(ng/mL, log-scale)** | |
|  | **N** | **Median (min-max)** | | **Wilcoxon p-value** | **Median (min-max)** | **Wilcoxon p-value** |
| **FIGO stage at diagnosis** |  |  | |  |  |  |
| Stage IIIc | 113 | 3.15 (1.54-5.27) | | 0.53 | 2.79 (2.13-4.85) | 0.38 |
| Stage IV | 35 | 3.12 (1.78-5.81) | |  | 2.86 (2.04-5.36) |  |
| **Histological subtype** |  |  | |  |  |  |
| Serous/papillary + grade 3 | 94 | 3.11 (1.80-5.81) | 0.57 | | 2.81 (2.04-5.36) | 0.36 |
| Other grade/histology | 24 | 3.33 (1.54-5.20) |  |  | 2.90 (2.08-4.61) |  |
| **Sugarbaker peritoneal Carcinomatosis Index** |  |  |  | |  |  |
| < 10 | 14 | 2.84 (1.54-4.13) | 0.12 | | 2.62 (2.15-3.98) | 0.19 |
| ≥ 10 | 127 | 3.14 (1.78-5.81) |  |  | 2.82 (2.04-5.36) |  |
| **Treatment arm** |  |  |  | |  |  |
| Nintedanib (experimental) | 102 | 3.10 (1.54-5.27) | 0.25 | | 2.82 (2.04-4.60) | 0.38 |
| Placebo (control) | 46 | 3.21 (1.95-5.81) |  |  | 2.79 (2.26-5.36) |  |

### Supplementary Table S3. Typical parameter estimates from the final semi-mechanistic model for circulating H3K27Me3 nucleosomes

| ***N*=142 patients** | **Estimate** | **RSE* on  estimate (%)** | **BSV** (%CV)** | **RSE*** on BSV (%)** | **Shrinkage (%)** |
| --- | --- | --- | --- | --- | --- |
| **K (D^-1^)** | 0.018 | 57.0 | 172.9 | 25.4 | 65 |
| **KPROD1 (IU.mL^-1^.D^-1^)** | 0.957 | 1.7 | 1.8 | 40.6 | 97 |
| **KPROD2 (IU.mL^-1^.D^-1^)** | 3.584 | 43.9 | 141.4 | 12.8 | 34 |
| **E50 (A.U.)** | 0.023 | 45.3 | 34.8 | 24.0 | 88 |
| **KELIM (D^-1^)** | 0.097 | 7.1 | 22.4 | 19.4 | 55 |
| **Nuc_0_ (IU.mL^-1^)** | 25.32 | 6.6 | 61.7 | 12.0 | 25 |
| **W** | 0.474 | 9.8 | - | - | - |

CV, coefficient of variation; BSV, between subject variability; K, treatment kinetic rate constant; KELIM, elimination rate constant K; KPROD, nucleosome tumor production rate constant; RSE, relative standard error

* Relative standard error calculated as (standard error/parameter) × 100.

** Between subject variability calculated as square root (var. random effect) × 100.

*** Percentage on standard deviation scale

### Supplementary Table S4. Typical parameter estimates from the final semi-mechanistic model for circulating H3K36Me3 nucleosomes

| *N* = 142 patients | Estimate | RSE* on estimate (%) | BSV** (%CV) | RSE*** on BSV (%) | Shrinkage (%) |
| --- | --- | --- | --- | --- | --- |
| K (D^-1^) | 0.009 | 71.4 | 210.0 | 26.4 | 63 |
| KPROD1 (IU.mL^-1^.D^-1^) | 1.952 | 2.5 | 1.4 | 219.9 | 96 |
| KPROD2 (IU.mL^-1^.D^-1^) | 3.605 | 44.5 | 132.3 | 20.1 | 39 |
| E50 (A.U.) | 0.014 | 20.9 | 12.5 | 50.6 | 95 |
| KELIM (D^-1^) | 0.184 | 1.3 | 2.0 | 140.9 | 94 |
| Nuc_0_ (IU.mL^-1^) | 19.190 | 5.5 | 51.6 | 11.4 | 23 |
| W | 0.370 | 12.8 | - | - | - |

CV, coefficient of variation; BSV, between subject variability; K, treatment kinetic rate constant; KELIM, elimination rate constant K; KPROD, nucleosome tumor production rate constant; RSE, relative standard error.

* Relative standard error calculated as (standard error/parameter) × 100.

** Between subject variability calculated as square root (var. random effect) × 100.

*** Percentage on standard deviation scale

### Supplementary Table S5. Univariable logistic prediction of the probability of obtaining a complete interval cytoreductive surgery

| **Variable** | **n** | **OR** | **95% CI** | **P** |
| --- | --- | --- | --- | --- |
| **Std H3K27Me3 KELIM (0.09)** | 139 | 4.37 | 0.22-109.46 | 0.34 |
| **Std H3K27Me3 KPROD1** | 139 | 0 | 0-inf | 0.33 |
| **Std H3K27Me3 KPROD2 (ROC)** | 139 | 1.04 | 0.87-1.26 | 0.65 |
| **Std H3K27Me3 KPROD2 (MAXS)** | 139 | 1.02 | 0.93-1.13 | 0.65 |
| **Std H3K27Me3 KPROD2 (Median)** | 139 | 1.05 | 0.85-1.33 | 0.65 |
| **Baseline H3K27Me3 (log-scale)** | 145 | 0.55 | 0.35-0.85 | 0.008 |
| **Pre-surgery H3K27Me3 (log-scale)** | 101 | 0.80 | 0.40-1.58 | 0.51 |
| **% change H3K27Me3 (log-scale)** | 80 | 1.00 | 0.99-1.02 | 0.66 |
| **Baseline H3K36Me3 (log-scale)** | 145 | 0.50 | 0.29-0.83 | 0.009 |
| **Pre-surgery H3K36Me3 (log-scale)** | 101 | 0.46 | 0.13-1.52 | 0.20 |
| **% change H3K36 (log-scale)** | 80 | 1.01 | 0.98-1.04 | 0.47 |
| **Std CA-125 KELIM (0.05)** | 131 | 7.29 | 3.37-17.13 | <0.0001 |
| **Baseline PCI (Sugarbaker)** |  |  |  |  |
| **<10** | 17 | REF | REF | REF |
| **≥10** | 157 | 0.51 | 0.17-1.41 | 0.21 |
| **HRD/BRCA status** |  |  |  |  |
| **BRCAwt/HRD negative** | 47 | REF | REF | REF |
| **BRCAwt/HRD positive** | 28 | 1.24 | 0.48-3.19 | 0.65 |
| **BRCAmut** | 27 | 2.94 | 1.10-8.39 | 0.035 |
| **Histology** |  |  |  |  |
| **Others** | 30 | REF | REF | REF |
| **Serous/papillary + grade 3** | 120 | 0.79 | 0.35-1.77 | 0.56 |
| **FIGO stage at diagnosis** |  |  |  |  |
| **IIIC** | 141 | REF | REF | REF |
| **IV** | 41 | 0.91 | 0.45-1.83 | 0.80 |
| **Response to NACT** |  |  |  |  |
| **Stable or progression** | 92 | REF | REF | REF |
| **Complete or partial response** | 71 | 1.78 | 0.95-3.36 | 0.07 |

BRCAmut, BRCA mutated; BRCAwt, BRCA wild type; CI, confidence interval; REF, reference; ROC, receiver operating characteristic; KELIM, elimination rate constant K; HRD, homologous recombination deficiency; NACT, neoadjuvant chemotherapy; OR, odds ratio; MAXS, maximally selected rank statistics; PCI, peritoneal carcinomatosis index; std, standardized

### Supplementary Table S6. Cox regression models of progression-free survival.

### Data derived from n=174 patients with n=146 events over a median of 11.9 (10.4-14.2) months

| **Variable** | **n** | **Events** | **Median** | **95% CI** | **Log-rank test** | **C-Index [95% CI]** | **Cox HR** | **95% CI** | **P** |
| --- | --- | --- | --- | --- | --- | --- | --- | --- | --- |
| ***H3K27Me3 KELIM score*** | | | | | | | | | |
| Unfavorable score - KELIM < cut-off | 39 | 35 | 11.2 | 8.5-16.0 | 0.30 | 0.53 [0.48-0.57] | REF | REF | 0.33 |
| Favorable score - KELIM ≥ cut-off | 101 | 83 | 12.9 | 10.9-15.5 |  |  | 0.82 | 0.55-1.22 |  |
| ***H3K27Me3 KPROD1 score*** | | | | | | | | | |
| Unfavorable score - KPROD1 ≥ cut-off | 70 | 60 | 11.1 | 9.3-15.1 | 0.60 | 0.52 [0.47-0.57] | REF | REF | 0.58 |
| Favorable score - KPROD1 < cut-off | 70 | 58 | 13.3 | 11.4-17.2 |  |  | 0.90 | 0.63-1.30 |  |
| ***H3K27Me3 KPROD2 score => MAXS*** | | | | | | | | | |
| Unfavorable score - KPROD2 ≥ cut-off | 127 | 110 | 11.6 | 10.4-14.4 | 0.08 | 0.51 [0.48-0.55] | REF | REF | 0.09 |
| Favorable score - KPROD2 < cut-off | 13 | 8 | 19.2 | 8.3-NR |  |  | 0.53 | 0.26-1.10 |  |
| ***Baseline H3K27Me3 (log-scale), MAXS (3.79)*** | | | | | | | | | |
| ≥ cut-off | 35 | 31 | 8.5 | 7.2-11.4 | 0.007 | 0.56 [0.52-0.60] | REF | REF | 0.008 |
| < cut-off | 105 | 86 | 14.4 | 11.2-17.2 |  |  | 0;57 | 0;38-0;87 |  |
| ***% change H3K27Me3 (log-scale), MAXS*** | | | | | | | | | |
| < cut-off | 53 | 44 | 11.3 | 9.1-15.5 | 0.30 | 0.54 [0.47-0.60] | REF | REF | 0.25 |
| ≥ cut-off | 29 | 22 | 17.2 | 10.4-31.1 |  |  | 0.74 | 0.44-1.24 |  |
| ***Baseline H3K36Me3 (log-scale), MAXS (2.45)*** | | | | | | | | | |
| ≥ cut-off | 113 | 98 | 10.9 | 9.6-13.5 | 0.03 | 0.55 [0.51-0.58] | REF | REF | 0.0317 |
| < cut-off | 27 | 19 | 18.5 | 12.9-NR |  |  | 0.58 | 0.36-0.95 |  |
| ***% change H3K36Me3 (log-scale), MAXS*** | | | | | | | | | |
| < cut-off | 38 | 32 | 9.3 | 8.4-15.3 | 0.05 | 0.57 [0.51-0.64] | REF | REF | 0.0552 |
| ≥ cut-off | 44 | 34 | 16.0 | 11.3-25.9 |  |  | 0.62 | 0.38-1.01 |  |
| CI, confidence interval; HR, hazard ratio; MAXS, maximally selected rank statistics; NR, not reached; REF, reference | | | | | | | | | |

### Supplementary Figure S1: Goodness-of-fit plots for circulating H3K27Me3 nucleosome modeling: observations vs individual predictions of H3K27Me3 KELIM (left), normal density over normalized prediction distribution errors (NPDE) histogram (middle), with observations measured in u/mL on a log-scale, and Visual Predictive Check (500 simulations) (right). Red lines represent Q10, Q50 (median) and Q90; area the 95% confidence interval and orange lines the binning intervals

###
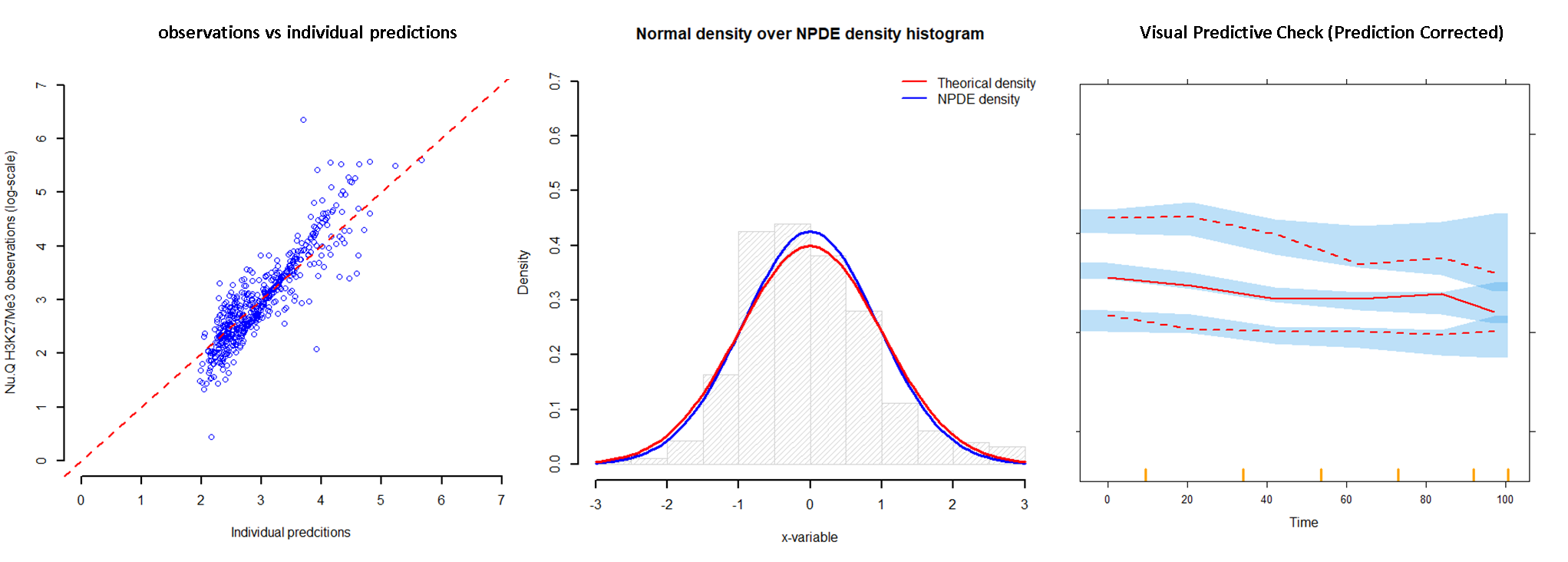


### Supplementary Figure S2. Goodness-of-fit plots for circulating H3K36Me3 modeling: observations vs individual predictions of H3K27Me3 KELIM (left), normal density over normalized prediction distribution errors (NPDE) histogram (middle), with observations measured in u/mL on a log-scale, and Visual Predictive Check (500 simulations) (right). Red lines represent Q10, Q50 (median) and Q90; area the 95% confidence interval and orange lines the binning intervals

**
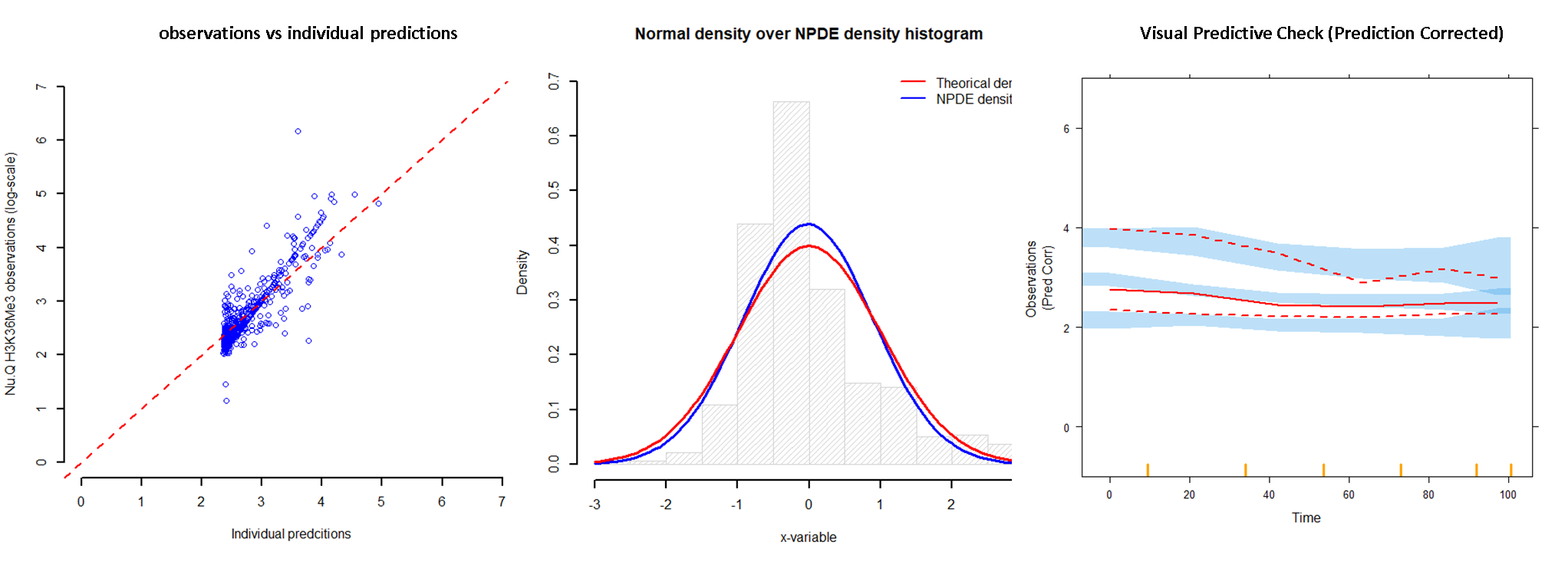
**
